# Does Subspecialization Matter? Institutional Outcomes Before and After Recruitment of Colorectal Surgeons

**DOI:** 10.1002/wjs.70544

**Published:** 2026-08-27

**Authors:** Daniel C. Steinemann

**Affiliations:** ^1^ Clarunis University Digestive Health Center University Hospital Basel Basel Switzerland; ^2^ Faculty of Medicine University of Basel Basel Switzerland

Over the past 2 decades, subspecialization within general surgery has become increasingly common. Although subspecialization in hepatopancreatobiliary (HPB) surgery is now widely established, the role of dedicated colorectal surgeons remains less universally accepted. Colorectal surgery encompasses many commonly performed procedures and is therefore often perceived as the domain of the general surgeon. However, the field has evolved considerably. The introduction of minimally invasive techniques and the integration of surgery into increasingly sophisticated multimodal treatment strategies have made both decision‐making and operative management substantially more complex. In the United States there are approximately 2000 board‐certified colorectal surgeons. The official board examination in this specialty was in established 1949 by the American Board of Colon and Rectal Surgery (originally founded as the American Board of Proctology). In Europe, over the past 25 years, approximately 750 surgeons have obtained board certification in coloproctology [1], a subspecialty of visceral or general surgery. In their current article, Kupietzky et al. analyze changes in the quality and volume of their colorectal program following the recruitment of two board‐certified colorectal surgeons in Israel [2].

Their principal findings include a 30% increase in lymph node yield, a higher proportion of minimally invasive procedures, and faster postoperative recovery of bowel function. In addition, operative volume increased substantially, and the institution began treating a greater proportion of complex cases. Previous large database analyses from the United States by Saraidaridis and, subsequently, Perdy et al. demonstrated that colectomies performed by general surgeons were associated with higher complication rates and mortality than those performed by colorectal surgeons. Importantly, this association persisted even after adjustment for both hospital and surgeon case volume [3, 4].

Considering the operative volume reported in this study, together with the relatively limited number of board‐certified colorectal surgeons overall, it is evident that many colorectal procedures continue to be performed by general surgeons. Nevertheless, the present study demonstrates that both the quality of care and the complexity of cases treated can evolve following the establishment of a dedicated colorectal service. An important question therefore remains: does subspecialization itself improve patient outcomes, or are the observed benefits primarily the result of broader institutional changes that accompany the development of specialized colorectal programs?

It is inherently difficult to attribute improvements in surgical outcomes to a single factor such as formal subspecialization. During the 7‐year study period alone, operative techniques and perioperative management changed considerably. Enhanced recovery after surgery (ERAS) pathways became increasingly standardized, minimally invasive surgery expanded substantially, perioperative care improved, and multidisciplinary management became routine. Within minimally invasive colorectal surgery, numerous techniques emerged over the past 2 decades, while others—including single‐port laparoscopy and transanal total mesorectal excision (TaTME)—have largely fallen out of favor. More recently, robotic surgery has become increasingly established. Similarly, complete mesocolic excision (CME) for right‐sided colon cancer, total neoadjuvant therapy, and nonoperative management strategies for rectal cancer have all been introduced into clinical practice.

Maintaining a high level of adherence to ERAS protocols is an ongoing process that requires continuous monitoring and close interprofessional collaboration. Likewise, multidisciplinary meetings for colorectal cancer, inflammatory bowel disease, and pelvic floor disorders have become an integral part of modern colorectal practice. All of these developments require dedicated leadership, sustained commitment, and effective organizational structures. This, in my view, is where the true value of the colorectal surgeon lies—not only in technical expertise but also in driving the development of the colorectal program and ensuring that it remains current.

The colorectal surgeon assumes responsibility for organizing the program, developing standard operating procedures (SOPs), introducing new techniques, ensuring quality assurance, contributing to academic development, and providing professional leadership and supervision within the general surgery team. Consequently, the institutional impact of subspecialization likely extends far beyond the technical performance of individual surgeons. Kupietzky et al. elegantly demonstrate that integrating board‐certified colorectal surgeons into the surgical team and entrusting them with leadership of the subspecialty strengthened the entire program [2]. Their institution became capable of attracting more complex referrals while delivering higher‐quality and less invasive surgical care.

In conclusion, although the available case volume and financial resources may not allow every institution to allocate all colorectal surgery exclusively to dedicated specialists, incorporating formally trained colorectal surgeons into a general surgery department appears to provide substantial benefits that extend beyond the operating room. Their impact encompasses not only individual patient care but also program development, quality improvement, education, and the long‐term evolution of colorectal services.

## Author Contributions

**Daniel C. Steinemann:** conceptualization, investigation, writing – original draft, methodology, validation, writing – review and editing.

## Funding

The author has nothing to report.

## Conflicts of Interest

The author declares no conflicts of interest.

## Data Availability

Data sharing not applicable to this article as no datasets were generated or analyzed during the current study.
